# Observation of magnetic skyrmions in unpatterned symmetric multilayers at room temperature and zero magnetic field

**DOI:** 10.1038/s41598-019-40705-4

**Published:** 2019-03-11

**Authors:** J. Brandão, D. A. Dugato, R. L. Seeger, J. C. Denardin, T. J. A. Mori, J. C. Cezar

**Affiliations:** 1 Laboratório Nacional de Luz Síncrotron, Centro Nacional de Pesquisa em Energia e Materiais, 13083-970 Campinas, SP Brazil; 2 Departamento de Física, Universidade Federal de Santa Maria, 97105-900 Santa Maria, RS Brazil; 3 Departamento de Física and CEDENNA, Universidad de Santiago de Chile, 9170124 Santiago, Chile

## Abstract

Magnetic skyrmions are promising candidates for the next generation of spintronic devices due to their small size and topologically protected structure. One challenge for using these magnetic states in applications lies on controlling the nucleation process and stabilization that usually requires an external force. Here, we report on the evidence of skyrmions in unpatterned symmetric Pd/Co/Pd multilayers at room temperature without prior application of neither electric current nor magnetic field. Decreasing the ferromagnetic interlayer thickness, the tuning of the physical properties across the ferromagnetic/non-magnetic interface gives rise to a transition from worm like domains patterns to isolated skyrmions as demonstrated by magnetic force microscopy. On the direct comparison of the measured and simulated skyrmions size, the interfacial Dzyaloshinskii-Moriya interaction (iDMI) was estimated, reveling that isolated skyrmions are just stabilized at zero magnetic field taking into account non-null values of iDMI. Our findings provide new insights towards the use of stabilized skyrmions for room temperature devices in nominally symmetric multilayers.

## Introduction

The full control of the magnetization processes in small-sized magnetic states is essential to increase the density of information in magnetic memories^1–3^. Magnetic skyrmions, i.e. nanometer-sized topological defects presenting swirling spin textures, are promising candi- dates for achieving efficiency and functionality towards room temperature devices^4–8^. Cur- rently, the standard heterostructures for studying skyrmions are heavy metal/ferromagnetic (HM/FM) interfacial systems, with structural inversion asymmetry in ultrathin films fabricated by magnetron sputtering. The strong spin orbit coupling (SOC) of the HM layer can lead to an antisymmetric exchange known as interfacial Dzyaloshinskii-Moriya interaction (iDMI)^9,10^, which plays a key role in the stabilization of chiral spins textures such as skyrmions^11–13^. The iDMI emerges in HM/FM interfaces owing broken spatial inversion symmetry which determines its sign and direction, whilst the iDMI magnitude depends on the SOC^14–16^. In this sense, different combinations of HM/FM interfaces have been investigated to obtain strong iDMI amplitudes and distinct signs to stabilize skyrmions and define their chirality^17–19^. Although isolated skyrmions have been observed recently at room temperature in this kind of systems, their nucleation and stabilization in most cases require external magnetic field and/or electrical current^20–22^. It would be interesting also to nucleate skyrmions without the need of any external force (magnetic field or current). This has been obtained using lithographically made structures to confine single or multiple skyrmions depending on the geometric parameters of the patterned samples^23,24^. It remains to be demonstrated that one can observe the spontaneous formation of skyrmions, even without nanostructured confinement.

In this work, we demonstrate that magnetic skyrmions can be stabilized at room temperature in unpatterned samples without the need for any preceding external excitation nor geometric confinement. We observed the formation of skyrmions in both as-grown (not exposed to magnetic field) and remnant states of nominally symmetric Pd/Co/Pd multilayers when the Co thickness is as thin as the percolation threshold of a continuous layer. The tuning of the magnetic properties at the HM/FM interfaces by simply thinning the FM layer leads to a transition of the magnetic domains pattern from a labyrinthine state, pass- ing through a state with long and separated stripes, then reaching isolated skyrmions for the lowest Co thickness. The observation of skyrmions was investigated mainly by magnetic force microscopy (MFM). We verified their reproducibility and density along the unpatterned thin film by imaging different regions of the sample to obtain the skyrmions average size. Besides, magnetization curves showed the dependence of the perpendicular magnetic anisotropy (PMA) and remnant magnetization with the Co thickness, providing a direct link to understand the magnetic textures observed in the MFM images. Micromagnetic simula- tions were carried out to elucidate the role of each magnetic parameter upon the skyrmions stability at room temperature and zero external magnetic field.

**Figure 1:**
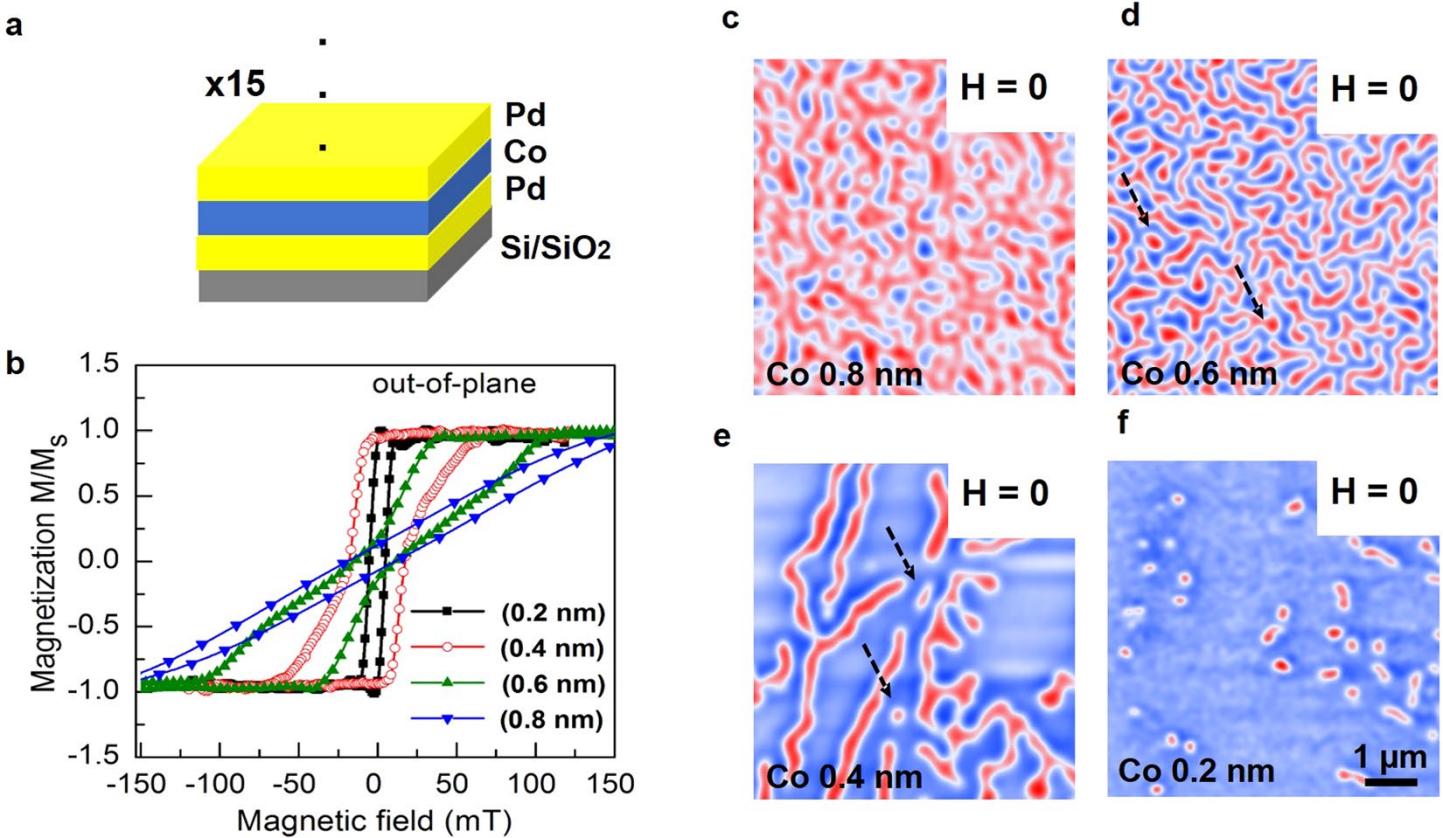
Representative magnetic multilayer, magnetization curves and MFM images. (**a**) design of the symmetric multilayer stack with the repetitions number. (**b**) hysteresis curves evolve from tail-like to square loop for decreasing Co thicknesses. (**c**–**f**) the MFM images acquired for different Co thicknesses in the as-grown state revealing the transition from small up and down domains, to isolated skyrmions. Red and blue contrasts represent the out-of-plane magnetization.

We prepared twin samples in the same sputtering runs (see methods), in order to measure hysteresis loops and take MFM images at zero magnetic field. Representative structure of the multilayer can be seen in Fig. 1a with the symmetric Pd/Co/Pd tri-layers and the repetitions number. As the Pd under and over layers nominally have the same thickness, we state our heterostructure as symmetric. We justify the repetitions number to enhance the magnetic contrast across the interface allowing the observation of the magnetic textures by means of MFM. The out-of-plane magnetization curves (normalized to the saturation magnetization *M*_*s*_) are summarized in Fig. 1b. For thicker Co samples (0.8 and 0.6 nm) the hysteresis show a tail feature, whilst thinner (0.4 and 0.2 nm) present a more square-shaped format. In-plane hysteresis loops were also performed (see Supplementary Information S1). To image the magnetic domains patterns without any prior applied magnetic field, the samples were imaged in the as grown state. For Co (0.8 and 0.6 nm), the MFM images show that the magnetization is broken in small domains (Fig. 1c,d). More specifically, for Co (0.6 nm) the magnetic domains exhibit a clear worm-like configuration in the so-called labyrinthine state (Fig. 1d). Reducing the Co thickness to 0.4 nm, the magnetic domain pattern shows long and separated stripes (Fig. 1e). It is very interesting that some skyrmions are clearly observed among both the worm-like and the long stripes patterns discussed so far, see the indications by dashed black arrows in Fig. 1d,e. In a previous work, similar skyrmions among the worm-like domain patterns were observed by MFM images^25^.

By further thinning the Co layer, the transition from worm-like to long stripes evolves to a new magnetic domain pattern for the thinnest Co (0.2 nm). As it is shown in Fig. 1f, many isolated skyrmions are observed. As the measurements were performed before cycling magnetic field, these images reveal that no prior stabilizing magnetic field or injection current are required to generate isolated skyrmions. Therefore, skyrmions at zero field can be spontaneously stable even for samples in the as-grown state.

To understand the sample properties that lead to the spontaneous formation of skyrmions at zero magnetic field, we refer to the hysteresis loop measured for one twin Co (0.2 nm) sample. Figure 1a shows that this sample has the most square-shaped loop and presents the highest remnant state and PMA. Thus, at zero magnetic field, owing high remanence and PMA, the magnetic pattern would be more favorable oriented out-of-plane in a uniform magnetization state. In our MFM images, skyrmions appear before reaching this ground state. This suggests that carefully controlling the transition from worm-like to single domain by varying the Co interlayer thickness, is feasible to create isolated stabilized skyrmions. Therefore, the combination of PMA and remnant magnetization emerges as one of the main sources for the generation of skyrmions at room temperature and zero magnetic field in Pd/Co/Pd symmetric multilayers.

On the other hand, it is well known that skyrmions are stabilized by the presence of interfacial Dzyaloshinskii-Moriya interaction (iDMI)^17^, which occurs in systems with structural inversion asymmetry^26,27^. Here, ideal symmetric Pd/Co/Pd multilayers should not have structural inversion asymmetry, so both Co/Pd and Pd/Co interfaces should contribute with iDMIs of same amplitude but opposite signs, hence leading to a null liquid iDMI strength. However, it has been observed that crystallographic asymmetry between Pt/Co and Co/Pt interfaces in Pt/Co/Pt systems can give rise to liquid iDMI^28^. Also, the total magnetic moment induced in the Pd is larger at the top Co/Pd interface than at the bottom Pd/Co, leading to an asymmetric magnetic proximity effect in Pd/Co/Pd trilayers^29^. This be- havior may also contribute to non-null iDMI in symmetric HM/FM/HM systems as the proximity effect might have a correlation with iDMI as suggested in the ref.^30^. Further- more, iDMI has been confirmed as the origin of asymmetric domain wall creep measured in symmetric Pd/Co/Pd multilayers by means of polar Kerr images^31^. Supported by these arguments, we consider that our symmetric Pd/Co/Pd multilayers may also present weak iDMI. Indeed, we will show through the correlation between micromagnetic simulations and MFM images that isolated skyrmions at zero magnetic field are just stabilized in symmetric multilayers considering a weak but no-null iDMI.

**Figure 2:**
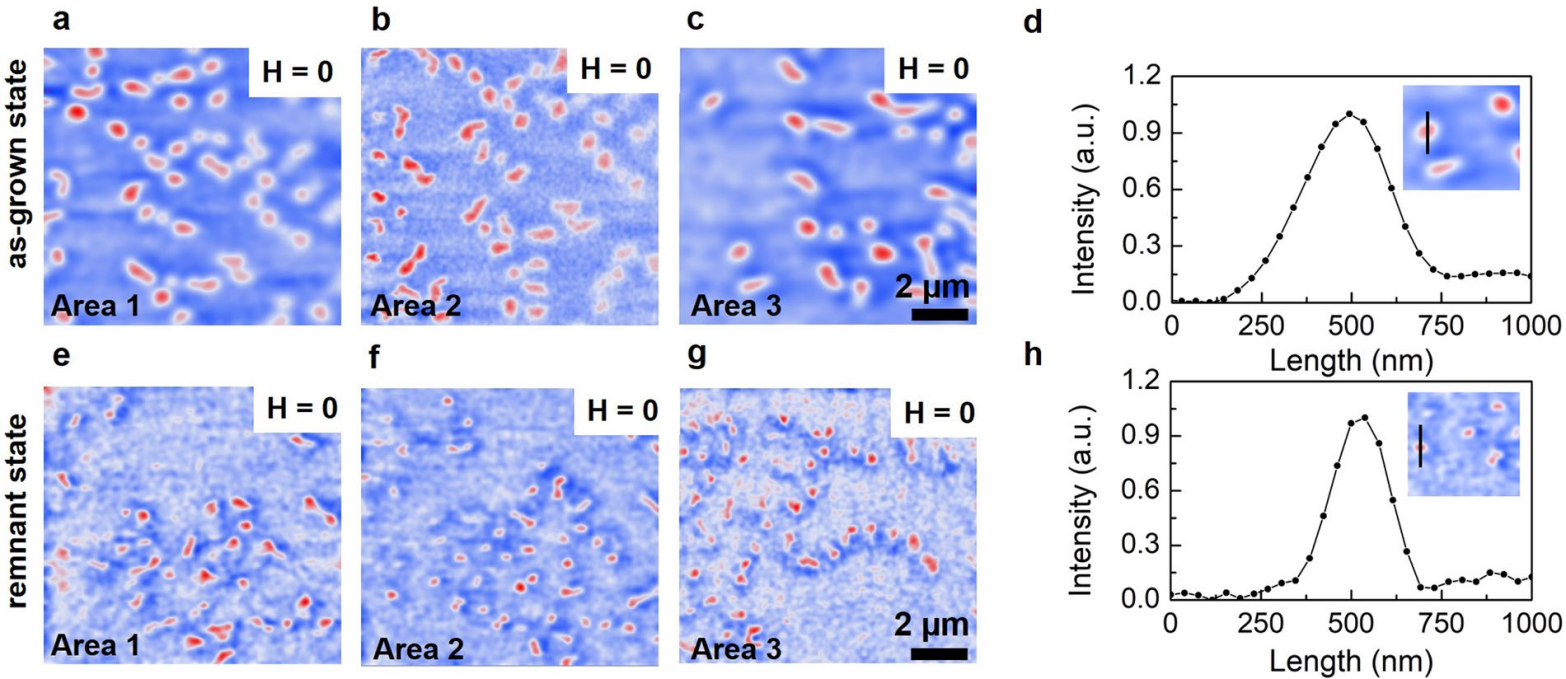
Representative MFM images of isolated skyrmions. (**a**–**c**) in the as-grown state. (**e**–**g**) in remnant state. Isolated skyrmions are observed at zero magnetic field over different places on the sample surface. (**d**,**h**) profile shows the circular-shaped feature of the isolated skyrmions. Insert, the representative line scan over one isolated skyrmion. Most notable, the skyrmions size is larger in the as-grown than in remnant state. MFM images obtained at different positions highlight the homogeneous formation of skyrmions.

A question that emerges here is whether the transition from worm-like to isolated skyrmions can be observed over the entire unpatterned sample. To verify this point, several MFM images were acquired on different regions of each sample (see methods). To illustrate, 3 different images are shown in Fig. 2a–c. All of them exhibit many isolated skyrmions, strengthening that their spontaneous formation is not an isolated case but rather is repro- ducible over different areas of the sample surface.

So far we demonstrated the observation of magnetic skyrmions at room temperature without any prior applied magnetic field. In order to explore the different processes that can stabilize skyrmions at zero magnetic field, the sample was imaged after applying and turning off an out-of-plane magnetic field. In the following we refer this as remnant state. The images of the samples Co (0.8 nm, 0.6 and 0.4 nm) (see Supplementary Information S2), present worm-like and long stripe patterns with a few isolated skyrmions. The same tendency of similar magnetic features was observed for the thinnest Co (0.2 nm), whose isolated skyrmions can be visualized in Fig. 2e–g. Similarly to the as-grown state images, these 3 MFM measurements are representative of the magnetic domains patterns over the entire unpatterned sample and proves that skyrmions can be generated and stabilized in symmetric Pd/Co/Pd multilayers in its remnant state after magnetization cycles. Notwithstanding, the isolated skyrmions presented in the Fig. 2 are randomly distributed and their size and shape are not identical. We suggest that this distribution arises from minor structural and morphological inhomogeneities along the different regions of the film as the Co layers are ultrathin. Besides, the variation of magnetic parameters such as *M*_*s*_, PMA, and iDMI along the polycrystalline sample also contributes to the different size and shape of stabilized skyrmions^32,33^.

Exploring the formation of skyrmions for the Co (0.2 nm) sample, we obtained their average size and density distribution for both as-grown and remnant states by analyzing 10 × 10 *µ*m^2^ MFM images from 10 different regions on the film. We used the full width at half maximum (FWHM)^34^, extracted from the line scan of 5 isolated skyrmion at each image to determine its size. This is illustrated in the Fig. 2d,h for an isolated skyrmion in the as-grown and other in remnant state, respectively. Therefore, 50 skyrmions were analyzed in each state. The as-grown skyrmions present an average size of 314 nm with a standard deviation of 41 nm, and are distributed with an average density of ∼0.3 skyrmions per *µ*m^2^. The skyrmions detected in the remnant state, in turn, exhibit a reduced average size of ∼165 nm with a standard deviation of 32 nm and a density of 0.25 skyrmions per *µ*m^2^.

The difference in the skyrmions size and density after submitting the sample to magnetic field represents a reduction of ∼55% and ∼18%, respectively. This comes from the fact that after applying magnetic field, a new process in the skyrmions generation occurs, allowing the formation of smaller skyrmions. Furthermore, after cycling the magnetic field and returning it to zero, some of the skyrmions may be annihilated, which can reduce the skyrmions density.

We call the attention that further studies on the skyrmions formation at room temperature and zero field in the as-grown and remnant states in heterostructures is required. Thus, the features related to skyrmions size and density observed in these two states can be clarified. Nonetheless, this observation demonstrates that skyrmions in these samples are robust and stable, being reproducible over different areas of the sample. These skyrmions observed in larger areas in combination with patterned nanostructures can be used as a source to feed constrictions in technological applications^35^. The advantage would be the fact that they do not need any stabilizing field to transform magnetic domains in a worm-like configuration into skyrmions. Therefore, they can be manipulated with ultra-low density current which is beneficial to perform low-energy spintronics devices.

In order to understand the formation of stabilized skyrmions at zero magnetic field, micromagnetic simulations were carried out using Mumax^3^ code^36^ (see methods). The physical parameter extracted from the hysteresis loops was the magnetization saturation *M*_*s*_. More specifically, for Co (0.2 nm), the measured *M*_*s*_ was ∼280 kA/m. The value is consistent with previous reported on Pd/Co multilayers^37–39^, where *M*_*s*_ was divided by the volume of the magnetic multilayer. To extract the perpendicular magnetic anisotropy, we used ${K}_{u}^{eff}=\frac{1}{2}{\mu }_{0}{{M}_{s}}^{2}$, which gives ${K}_{u}^{eff}$ ≈ 0.06 MJ·m^−3^. In a recent work, effective perpendicular anisotropy ${K}_{u}^{eff}$ obtained for Co/Pd multilayes, similar values of PMA were obtained when the Pd layer is thicker than 0.9 nm^40,41^. We varied the magnetization saturation *M*_*s*_ and magnetic anisotropy ${K}_{u}^{eff}$ to understand the impact of these properties on the skyrmions stability considering a weak Dzyaloshinskii-Moriya below *D* = 0.8 mJ·m^−2^. The magnetic exchange stiffness *A*_*exch*_ = 15 pJ·m^−3^ and Gilbert damping *α* = 0.3 parameters were kept fixed.

**Figure 3:**
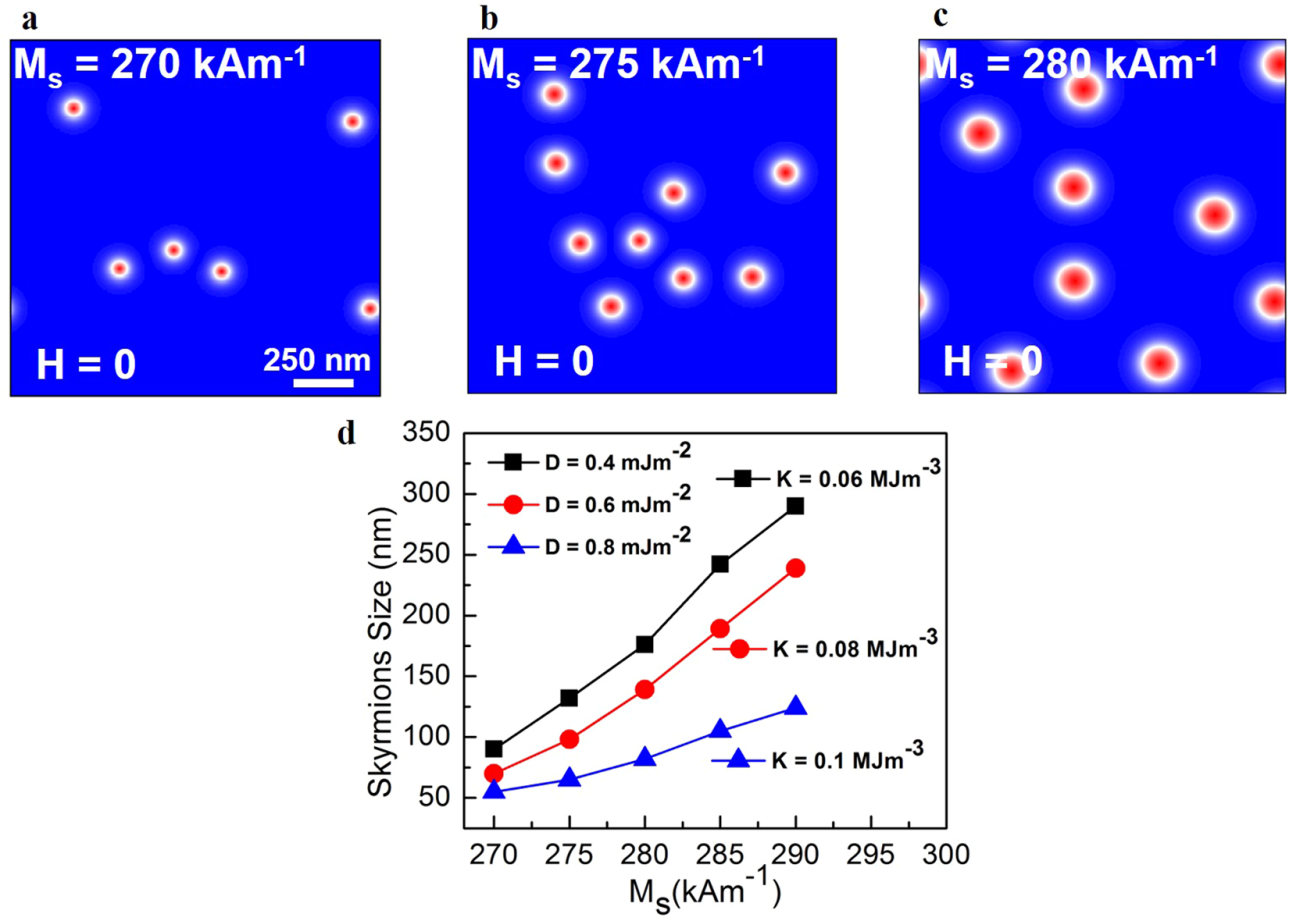
Simulated skyrmions at zero magnetic field for *D* = 0.4 mJ·m^−1^. (**a**–**c**) the skyrmions are isolated and randomly distributed as it was observed experimentally. The saturation magnetization parameter was varied showing that the skyrmions becomes larger as *M*_*s*_ increases. The scale bar for the images (**a**–**c**) is 250 nm. In (**d**) the skyrmions size extracted from the simulations to different D and ${K}_{u}^{eff}$ (labeled *K* in the figure). The skyrmions are larger when ${K}_{u}^{eff}$ and *D* are reduced for a ranging of *M*_*s*_ values.

We start by showing examples of micromagnetic simulations on the stabilized skyrmions at zero magnetic field considering a constant *D* = 0.4 mJ·m^−2^. Figure 3a–c, show isolated skyrmions when ${K}_{u}^{eff}$ is 0.06 MJ·m^−3^ and *M*_*s*_ ranging from 270 kA·m^−1^ to 280 kA·m^−1^. Most notable, the higher the *M*_*s*_ the larger the skyrmions size, suggesting that local variations in the magnetic properties (${K}_{u}^{eff}$, *M*_*s*_ and *D*) beyond the non-uniformity across the interfaces play also a role in modifying the skyrmions size. Indeed, this behavior is summarized in Fig. 3d. Varying *M*_*s*_, we obtained simulated skyrmions size ranging from ∼50 nm to ∼290 nm considering different values of *D* and ${K}_{u}^{eff}$. It indicates that larger skyrmions are stabilized when both *D* and ${K}_{u}^{eff}$ are reduced while *M*_*s*_ is increased. The experimental and simulated results show a good agreement. This direct comparison on the skyrmions size, allowed us quantitatively estimate *D* around 0.4–0.6 mJ·m^−2^. Similar results were obtained by performing simulations using negative *D* values, (see Supplementary Information S3).

**Figure 4:**
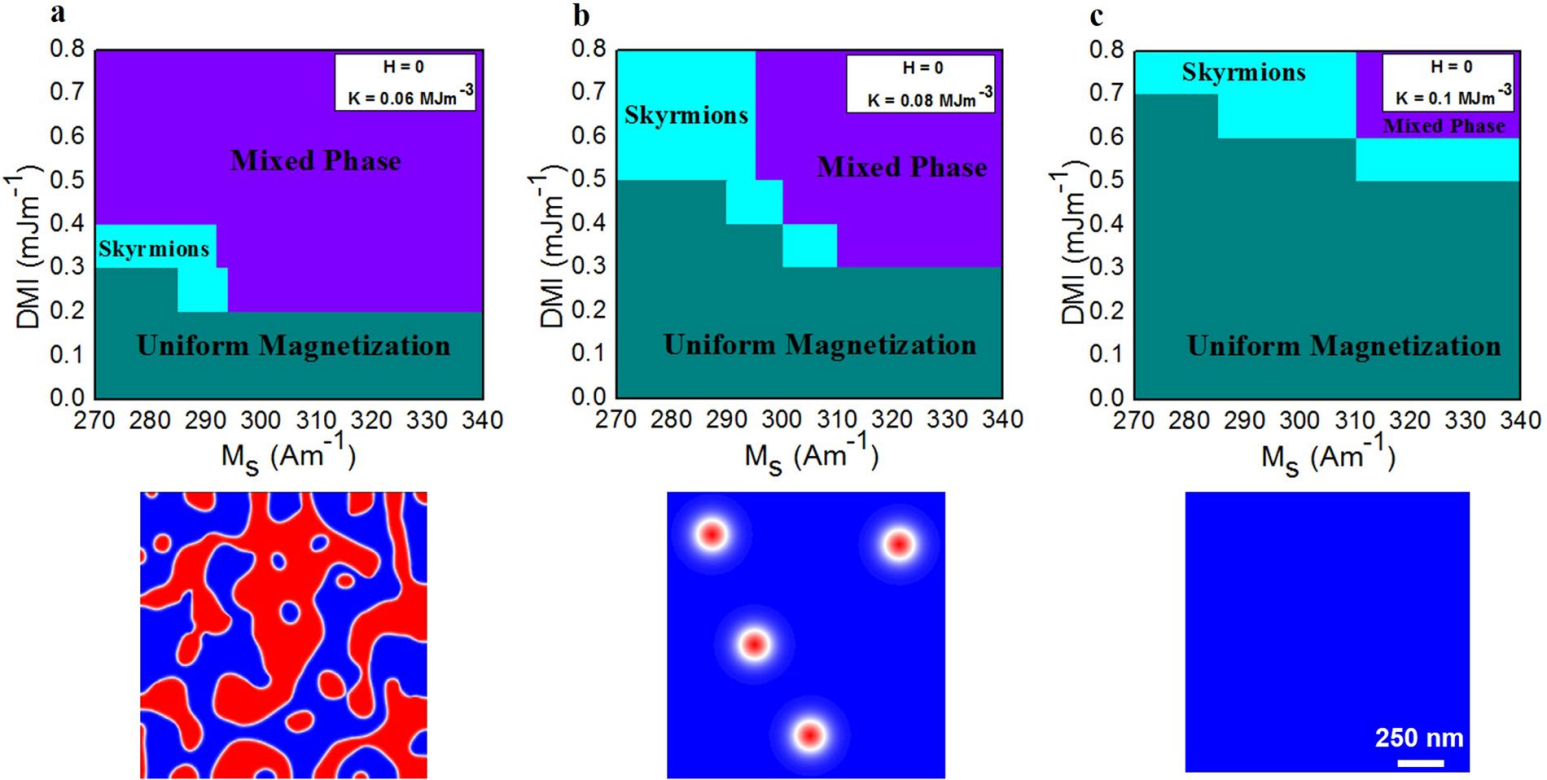
Simulated phase diagram. The ground state was determined by modifying D and *M*_*s*_ for different k. (**a**) below D = 0.2 mJ·m^−1^ the uniform magnetization pattern is the ground state. Above D = 0.2 mJ·m^−1^ isolated skyrmions are stabilized for *M*_*s*_ ranging from 270–295 kA·m^−1^ and a mixed phase (skyrmions and stripes) is stabilized for higher D. (**b**,**c**) show that these magnetic states are also observed when DMI becomes larger. Representative configurations with mixed phase, isolated skyrmions and uniform magnetization are also presented. The scale bar is 250 nm.

Finally, we performed further simulations to build up a phase diagram to explore the physical parameters needed to stabilize skyrmions at zero magnetic field. The simulated DMI *vs M*_*s*_ phase diagram is shown in Fig. 4. It distinguishes three different phases as the ground state. In Fig. 4a, the mixed phase, in which isolated skyrmions and magnetic stripes coexist, is more favorable independently of *M*_*s*_ when *D* is higher than 0.4 mJ·m^−1^. A representative configuration of this mixed phase is shown on the bottom of the Fig. 4a. Changing *D* between 0.2 and 0.4 mJ·m^−1^ for low values of *M*_*s*_, a narrow region is observed in which isolated skyrmions are stabilized. For lower *D* values below 0.2 mJ·m^−1^, the uniform magnetization is the ground state. Interestingly, increasing ${K}_{u}^{eff}$, the region where skyrmions are stable is wider, see Fig. 4b. A representative configuration of this stabilized skyrmions is shown on the bottom of the Fig. 4b. Besides, it is noted that the uniform magnetization pattern increases while the mixed phase is reduced. For higher ${K}_{u}^{eff}$ the isolated skyrmions are stabilized for larger *D*, see Fig. 4c. The mixed phase is drastically reduced, while the uniform magnetization is mostly observed as the ground state. A representative configuration of the uniform magnetization is shown on the bottom of the Fig. 4c. The diagram shows how the physical parameters can be tailored in order to achieve the desired magnetic pattern, which is an important mechanism for designing room-temperature skyrmions based devices.

In conclusion, we investigated the formation of magnetic domains patterns in Pd/Co/Pd multilayers using MFM images. By thinning the Co interlayer we observed a transition from worm-like magnetic domains pattern to isolated skyrmions at room temperature and zero magnetic field. Stabilized skyrmions were observed in samples in both as-grown and remnant states, and their homogeneity was proved by several MFM images taken over dif- ferent areas of the sample surface. Comparing the measured and simulated skyrmions size, the quantitative interfacial Dzyaloshinskii-Moriya interaction (iDMI) was estimated with D ranging from 0.4 to 0.6 mJ·m^−1^ as one of the key physical properties on the skyrmions stability. Symmetric multilayers grown on Si substrate by magnetron sputtering are very suitable to host skyrmions in unpatterned samples without any stabilizing field at room temperature, providing a new path towards skyrmions-based devices. They can be used in areas exceeding 10 × 10 *µ*m^2^, and eventually manipulated with ultra-low current density in order to perform devices combined with narrow constrictions without the need to transform magnetic worm-like domains in skyrmions.

## Methods

### Film deposition and magnetic characterization

The Pd/Co/Pd multilayers were grown onto Si substrates by magnetron sputtering at room temperature and deposition pressure of 2 mTorr in the argon atmosphere. Two iden- tical samples were grown in each sputtering process in order to acquire MFM images at zero magnetic field and also magnetization curves. To increase the magnetic contrast for MFM measurements and the signal-to-noise ratio for magnetic hysteresis curves, fifteen repetitions of the trilayers were grown. X-rays reflectometry (XRR) measurements were performed to verify the Pd and Co thicknesses. The magnetization reversal as a function of magnetic field was acquired by using an alternating gradient field magnetometer (AGFM) and vibrating- sample magnetometer (VSM).

### Magnetic Force Microscopy Images

MFM images were carried out using a Nanosurf FlexAFM microscope. We used MagneticMulti75-G MFM tips from Budget Sensors, they are coated by a cobalt alloy hav- ing magnetic moment of roughly 10^−16^ Am^2^ and coercivity of roughly 0.03 T. We operated the MFM measurements at the dynamic force mode with a resonant frequency of about 75 kHz. The images were acquired in the tip-surface distance of 60 nm. To acquire images in different areas, we moved the sample over distances of 1 mm in order to confirm the homogeneity of the domain magnetic patterns.

### Micromagnetic Modeling

Micromagnetic simulations were performed using the Mumax^3^ GPU-accelerated program over an area of 1.5 × 1.5 *µ*m^2^ discretized in cells size of 3 × 3 × 3 nm^3^. The magnetic anisotropy ${K}_{u}^{eff}$, magnetization saturation *M*_*s*_, and Dzyaloshinskii-Moriya value *D* were modified to understand their impact on the skyrmions stability. We experimentally obtained ${K}_{u}^{eff}$ from the magnetization measurements, which intrinsically includes the component due to the de- magnetizing field. Thus, to avoid taking this term twice in consideration we disabled the automatic shape anisotropy calculation in the micromagnetic simulations. To obtain the magnetic ground state, the initial magnetization is chosen randomly to seek for the same aleatory distribution of the experimental observed skyrmions. Subsequently, the magnetiza- tion is left to relax for 1 *µ*s, then by minimizing the energies involved, reach an equilibrium condition which represents the skyrmions stability. The long time 1 *µ*s, to carry out the simulations, was chosen in order to verify if the skyrmions would persist after being stabi- lized. No transformation after their stability was observed. To clarify the influence of the magnetic parameters in the stabilized skyrmions shown in the phase diagram, further simu- lations were done using negative values of *D*. Comparable results were obtained regarding the ferromagnetic order and skyrmions stability.

## Supplementary information

Observation of magnetic skyrmions in unpatterned symmetric multilayers at room temperature and zero magnetic field
