# Supplementary material for: Observation of magnetic skyrmions in unpatterned symmetric multilayers at room temperature and zero magnetic field
Source: Sci Rep. 2019 Mar 11;9:4144. doi: 10.1038/s41598-019-40705-4 (PMC6412027; doi:10.1038/s41598-019-40705-4)
Supplement: Supplementary file 1 — Observation of magnetic skyrmions in unpatterned symmetric multilayers at room temperature and zero magnetic field [file 41598_2019_40705_MOESM1_ESM.doc]

**Supplementary Information**

**Observation of magnetic skyrmions in unpatterned symmetric multilayers at room temperature and zero magnetic field**

**J. Brandão,1 D. A. Dugato,1, 2 R. L. Seeger,2 J. C. Denardin,2, 3 T. J. A. Mori,1 and J. C. Cezar1**

**^1^**Laboratório Nacional de Luz Síncrotron, Centro Nacional de Pesquisa em Energia e Materiais, 13083-970 Campinas SP, Brazil

**^2^**Departamento de Física, Universidade Federal de Santa Maria, 97105-900 Santa Maria RS, Brazil

**^3^**Departamento de Física and CEDENNA, Universidad de Santiago de Chile, 9170124 Santiago, Chile

**S1. Magnetization Reversal for in and out-of-plane magnetic field**

To determine the magnetization reversal and extract more information related to the magnetic properties of the [Pd/Co/Pd] multilayers, magnetization curves were measured for in-plane magnetic field. Figure 1 (a) and (b) show the magnetization reversal behavior in [Pd(2nm)/Co_(X)_/Pd( 2nm)]x15, for X = 0.6 and 0.8 nm. For Co (0.6 nm), the in-plane magnetization reversal presents a low remanence comparing to the out-of-plane magnetization curve, see Fig1. (a). Looking to the Fig1. (b), the in-plane and out-of-plane magnetization reversal shows almost equal behavior with low remanence. This behavior suggests that Co (0.8 nm) is the thicker thickness that can host perpendicular magnetic anisotropic (PMA) in Pd/Co/Pd multilayers, as it was already determined in previous work [1,2]. These results corroborate to the magnetic domain patterns observed at zero magnetic field in the MFM images in which the magnetization breaks in narrow magnetic domains with opposite directions.

The anisotropy field (see dashed arrows), which is obtained from the in-plane magnetization curve at saturation is higher for Co (0.6 nm) comparing to (0.8 nm). This larger anisotropy field for thinner Co thicknesses shows that the in-plane magnetic anisotropy (IMA) is reduced leading to the increased PMA. It was confirmed by in-plane magnetization curves measurements for Co (0.4 nm) and (0.2 nm). No magnetization reversal was obtained when the magnetic field as large as 2T was applied.


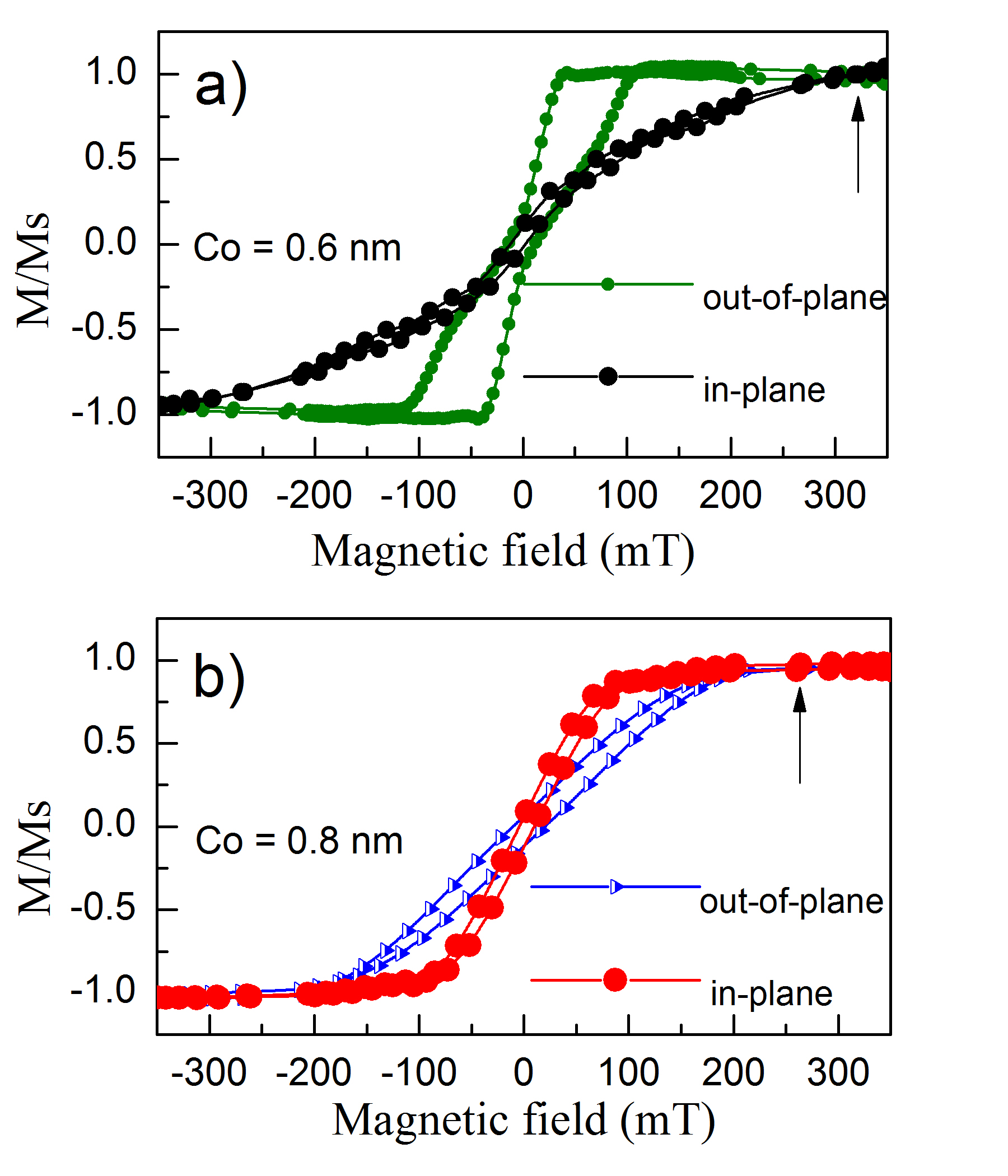


**Figure 1.** **Magnetization reversal obtained in Pd(2nm)/Co(_T)_/Pd(2nm)**. **a**, for Co (0.6 nm) the reversal switching for in-plane magnetic field shows low remanence. **b**, for Co (0.8 nm) similar behavior in the magnetization reversal was acquired for both in and out-of-plane magnetic fields.

**S2. Domains pattern imaged by magnetic force microscopy**

Further MFM images, not shown in the main text, were performed to observe the homogeneity on the magnetic domain patterns formation and skyrmions stability after submitted to the magnetic field. In the Figure 2 (a-d), we show examples of MFM imagens acquired in the remnant state.


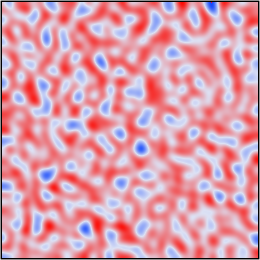

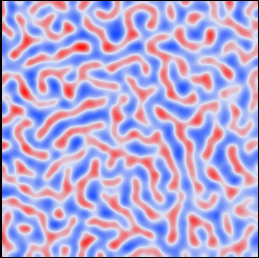


**Co 0.8 nm**

**Co 0.6 nm**

**(a)**

**(b)**


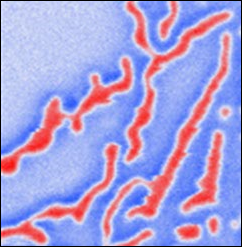


**Co 0.4 nm**


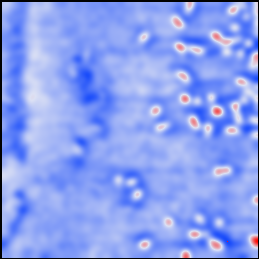


**(c)**

**(d)**

**Co 0.2 nm**

**1 µm**

**Figure 2.** **MFM imagens**. **a-c**, magnetic domain patterns. **d**, isolated skyrmions. The imagens were acquired at remnant state. The results show very similar behavior compared to ones observed in the as-grown state.

The magnetic domains patterns and isolated skyrmions show quite similar behavior compared to ones shown in the main text. A clear transition from small domains and worm like for thicker Co thicknees is replaced by long stripes for Co (0.4 nm), and finally isolated skyrmions for the thinest Co (0.2 nm) thickness. Even after applying the magnetic field the domains patterns and skyrmions survive at remnant state.

**S3. Micromagnetic Simulations Modelling**

As mentioned in the main text, futher simulations were carried out using values of iDMI lower than 0.8mJm^-2^. We look by performing these simulations for more insight on the skyrmions stability at zero magnetic field. The Figure. 3 shows examples of stabilized skyrmions obtained for Ms = 340 kAm^-1^, k = 0.1 MJm^-3^ and D = 0.6mJm^-2^.


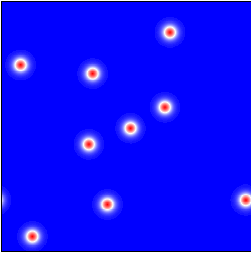

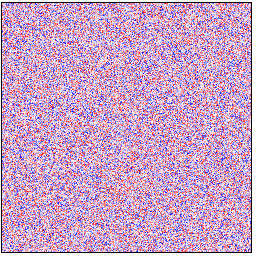

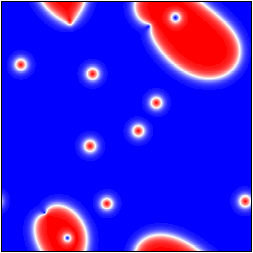

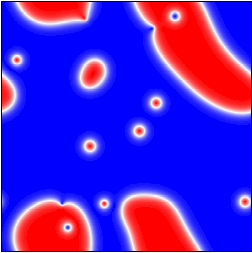

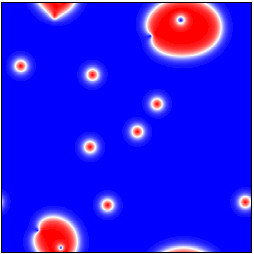

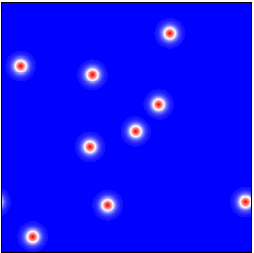

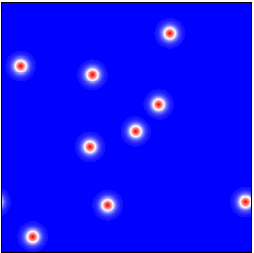

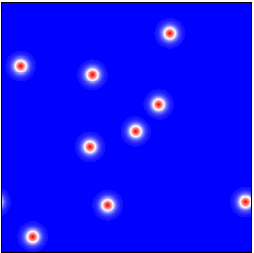


**Time = 0s**

**Time = 5 ns**

**Time = 10 ns**

**Time = 15 ns**

**Time = 25 ns**

**Time = 45 ns**

**Time = 90 ns**

r in size, see Figure.3 (d).

zed skyrmions ground state is shown in the Figure. DMI = e observed and they have two orientatio**Time = 105 ns**

**Figure 3.** **Simulated skyrmions generation**. The initial magnetic configuration is random. After 15 ns, small circular domains with both polarities are created. At 25 ns, isolated skyrmions with defined polarity is stabilized. Even at 105 ns the skyrmions still stabilized and they not change the shape or size.

We started the simulation at t = 0s, using a randomic magnetic configuration and then the magnetization is left to relax in order to find the ground state, in which represents the domain pattern. As it can be seen, after 5 ns the configuration shows regions with blue (- Mz) and red (+ Mz) contrasts. The Mz is the magnetic component out-of-plane. One can see that skyrmions are observed and they have core with two orientations (blue and red). At 10 and 15 ns, the blue contrast (- Mz) becomes larger as the red (+ Mz) is shrinking. At 25 ns, no long wider regions with red contrast can be seen anymore, and skyrmions are observed with the core polarization in the (+ Mz) orientation. These skyrmions maintain the same structure (shape, size, and position) at 105 ns. Even for long time 1000s ns, they are not anihillated or trasformed in any new configuration. We used this process to obtain the simulated stabilized skyrmions. In order to explore the role of Ms and k in the skyrmions stability for a lower D = 0.6 mJm^-2^, simulations were carried out by fixing k at 0.1 MJm^-3^varying Ms. The results are summarized in the Figure. 4.


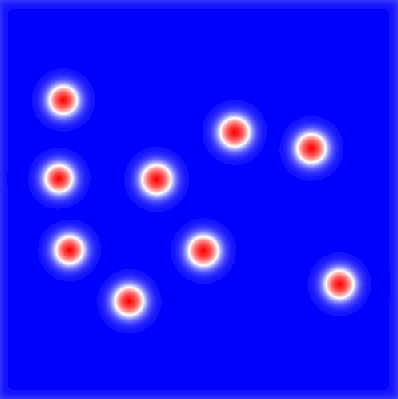


**M_s_= 350 KA/m**

**(d)**


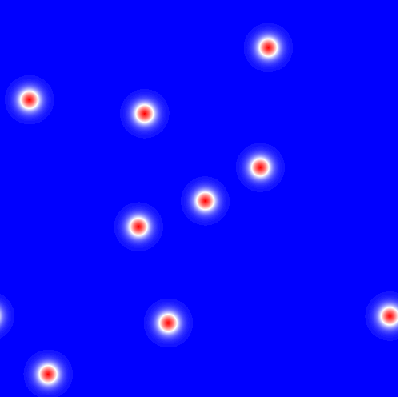


**M_s_= 340 KA/m**

**(c)**


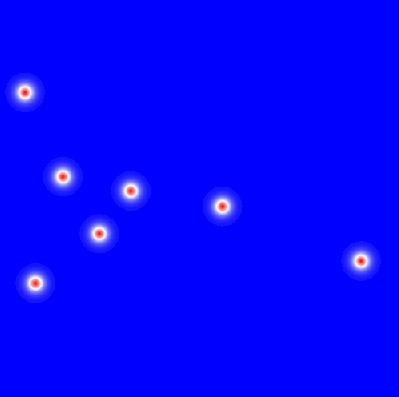


**(b)**

**M_s_= 330 KA/m**


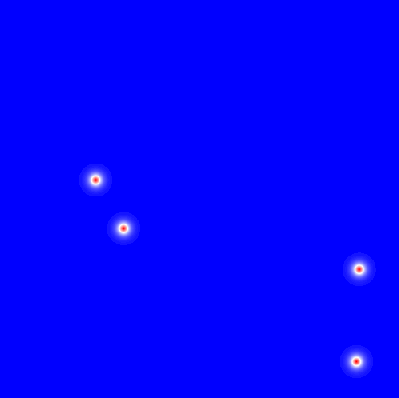


**(a)**

**M_s_= 320 KA/m**

**Figure 4.** **Simulated stabilized skyrmions.** The skyrmions are stabilized for higher values of Ms comparing to ones stabilized at D = 0.6 mJm^-2^.

As the representative stabilized skyrmions obtained from the micromagnetic simulations shown in the main text for D = 0.8 mJm^-2^, these simulated skyrmions for D = 0.6 mJm^-2^ have similar features. They are distributed randomily and their size depend of Ms strenght. Most notable, the simulated skyrmions for lower D are obtained for higher values of Ms. Furthermore, it is interisting that skyrmions for lower iDMI and higher Ms were found to be larger in size, see Figure 4 d.

To investigate the skyrmions type observed in the MFM measurements, micromagnetic simulations were also done by using negative DMI values as D = - 0.8 mJm^-2^ and D = - 0.6 mJm^-2^. The simulations show that Néel Skyrmions are stabilized at zero magnetic field. However, for positive DMI as demostrated in the main text, the spins point radially to the skyrmions core. Otherwise, for negative DMI the spins point radially out to the skyrmions core, which shows the influence of DMI sign in the skyrmions chirality, see Figure 5.


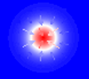

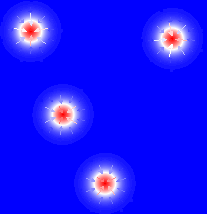

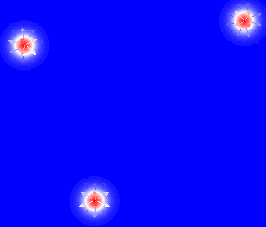

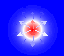


**Negative DMI**

**PositiveDMI**

**Figure 5.** **Stabilized skyrmions at negative DMI**. The Néel skyrmions show spins ponting radially out to the skyrmions core. The polarity for both positive and negative DMI points to the same up direction.

We show next additional simulations for a much smaller D = 0.3 mJm^-2^. These simulations were performed to understand the threshold which the skyrmions can be stabilized by a lower DMI. In this example, the magnetic parameters were beyond the iDMI, k = 0.1 MJm^-3^ and Ms = 300 kAm^-1^. The simulations shown in the Figure. 6 that, after initial random magnetic configuration, the magnetization evolves very faster for a single domain state, which is stabilzed after 22.5 ns. The uniform magnetization patter is therefore, mostly observed for lower iDMI.


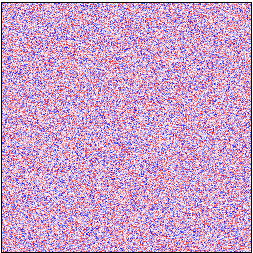

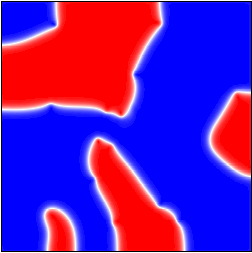

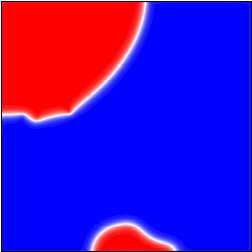

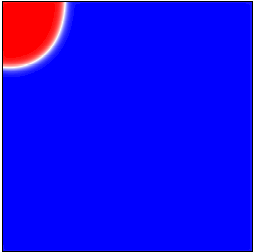

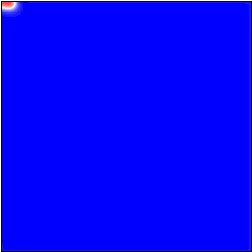

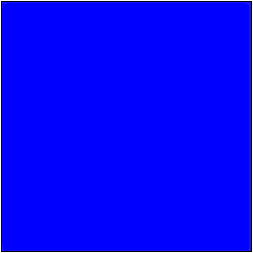


**0 ns**

**2.5 ns**

**10 ns**

**20 ns**

**22.5 ns**

**27.5 ns**

**(a)**

**(b)**

**(c)**

**(d)**

**(e)**

**(f)**

**Figure 6. Simulated ground state.** Obtained for lower D = 0.3 mJm^-2^. The simulations show that uniform magnetization pattern is preferentially stabilized.

References

[1] Z. Liu, R. Brandt, O. Hellwig, S. Florez, T. Thomson, B. Terris, and H. Schmidt,Thickness dependent magnetization dynamics of perpendicular anisotropy Co/Pd multilayer films. J. Magn. Magn. Mater. 323, 1623 (2011).

[2] Y. Kachlon et al., Extracting magnetic anisotropy energies in Co/Pd multilayers via refinement analysis of the full magnetoresistance curves. J. Appl. Phys. 115, 173911 (2014).
